# Insights into the Chemical Compositions and Health Promoting Effects of Wild Edible Mushroom *Chroogomphus rutilus*

**DOI:** 10.3390/nu15184030

**Published:** 2023-09-17

**Authors:** Bincheng Han, Jinhai Luo, Baojun Xu

**Affiliations:** 1 Food Science and Technology Program, Beijing Normal University-Hong Kong Baptist University United International College, Zhuhai 519087, China; 2 Centre for Cancer and Inflammation Research, School of Chinese Medicine, Hong Kong Baptist University, Hong Kong, China

**Keywords:** *Chroogomphus rutilus*, chemical compositions, health-promoting effects

## Abstract

*Chroogomphus rutilus* is an edible mushroom that has been an important food source since ancient times. It is increasingly sought after for its unique flavor and medicinal value. It is one of the most important wild mushrooms for its medicinal and economic value. *C. rutilus* contains a variety of active ingredients such as vitamins, proteins, minerals, polysaccharides, and phenolics. *C. rutilus* and its active compounds have significant anti-oxidant, anti-tumor, immunomodulatory, anti-fatigue, hypoglycemic, gastroprotective, hypolipemic, and neuronal protective properties. This paper summarizes the fungal chemical compositions and health-promoting effects of *C. rutilus* by collecting the literature on the role of *C. rutilus* through its active ingredients from websites such as Google Scholar, Scopus, PubMed, and Web of Science. Current research on *C. rutilus* is limited to the cellular and animal levels, and further clinical trials are needed to conduct and provide theoretical support for further development.

## 1. Introduction

Many health problems in today’s society hurt people’s quality of life and wellbeing. Some of these health problems include cardiovascular disease, obesity, diabetes, cancer, and immune system disorders. These problems are closely linked to modern lifestyles, dietary changes, and environmental factors [1]. Cardiovascular diseases, such as hypertension and hypercholesterolemia, have become major health problems worldwide [2]. Diets high in salt, sugar, and fat, physical inactivity, and stress can all contribute to the development of cardiovascular disease [3]. Obesity is also a growing problem that increases the risk of many non-communicable diseases not only related to cardiovascular disease, but also diabetes, joint disease, stroke, and other chronic diseases [4]. Cancer is one of the world’s greatest health challenges. Environmental factors, unhealthy lifestyles (e.g., smoking, alcohol consumption, etc.), dietary patterns, and genetic mutations can all increase the risk of cancer [5]. The dysregulation of the immune system is also a key factor in the development of many diseases, including autoimmune and infectious diseases [6]. In the face of these health problems, there is an increasing emphasis on proactive health management measures, including improving diet, increasing physical activity, reducing stress, and boosting immunity [7]. Diet plays an important role in health management [8]. The consumption of nutrient-rich, functional foods is gradually increasing, as is the demand for natural, organic, low-fat, high-fiber, and antioxidant-rich foods [9].

Since ancient times, mushrooms have been a critical element in human life and culture as a kind of food source. It is an important part of the human diet to promote health and wellbeing [10]. Traditional Chinese medicine also often uses mushrooms as medicinal products [11]. China is a major country in mushroom cultivation and production [12]. Moreover, Heilongjiang Province, Jilin Province, and Liaoning Province in China have a forest coverage rate of 80%, abundant annual rainfall, abundant natural conditions, and abundant sunshine, which provide unique growth conditions for wild mushrooms and are the main mushroom-producing areas [13]. Mushroom mycelium is considered as a nutritional drug [14]. The fruiting body of the mushroom includes the cap and stalk [15]. In the past decades, the knowledge about the bioactive components and nutritional value of mushrooms has been increasing. Mushrooms are considered to be rich sources of many bioactive compounds.

Mushrooms are rich in high-quality carbohydrates [16]. Their high-quality protein [17], polysaccharides [18], unsaturated fatty acids [19], minerals [20], sterols and secondary metabolites [19], and vitamin D [21]. The important role of mushrooms in protecting and treating various health problems has always been appreciated, and mushrooms play a huge role in treating various degenerative diseases [22]. Mushrooms also play a significant role in the treatment of immunodeficiency, cancer, inflammation, hypertension, hyperlipidemia, hypercholesterolemia, and obesity [18]. The bioactive components of Agaricus campestris have the anti-oxidation effect [23] and anti-tumor [24] effect, which serves as antioxidants and anti-tumor drugs.

*Chromogompuhus rutilus* (*Gomphidius viscidus*, *Gomphidius rutilus*), abbreviated as *C. rutilus* and also known as the brown slime cap [25], red mushroom, pine umbrella, pine mushroom, and the copper spike, is a member of the genus *Chromogompus* in the family *Gomphidiaceae*. *C. rutilus* belongs to the *Basidiomycota, Agaricales, and Gomphidiaceae* families [26]. *Chromogompus* was originally a subgenus of *Gomphidius* [27] and was elevated to the genus status by Orson K in 1964 [28]. Molecular analyses have shown that *Gomphidius* is monophyletic [29,30]. As an ectomycorrhizal fungus, *Chroogomphus rutilus* is symbiotic with *Pinus densiflora* in Europe and is often found with *Lactobacillus* under Chinese pine forests in China [31].

Therefore, mushrooms have attracted wide attention in the research area of food nutrition, and this research may provide a way to prevent and treat some chronic diseases. In this case, we aim to provide an overview and summary of the nutritional value, fungal–chemical profile, and biological activity of *C. rutilus*, which will provide better knowledge of health improvement. With further progress in the related fields, it may be that *C. rutilus* can play a more important role in medicine. This paper aims to summarize the fungal chemistry, medicinal nutritional value, and biological activity of *C. rutilus* by collecting the literature on its active compounds from Google Scholar, Scopus, PubMed, and Web of Science. The keyword for collecting journal article includes *Chroogomphus rutilus*, *C. rutilus*, and antioxidant mushrooms. It is proven that *C. rutilus* is an edible mushroom with remarkable antioxidant, anti-tumor, immunomodulatory, anti-fatigue, hypoglycemia, gastric protective, hypolipidemic, and neuronal protective effects. However, research on *C. rutilus* is currently limited to the cellular and animal levels, and further clinical trials are needed to provide theoretical support for further development.

## 2. Description and Geographical Distribution of *C. rutilus*

**Figure 1:**
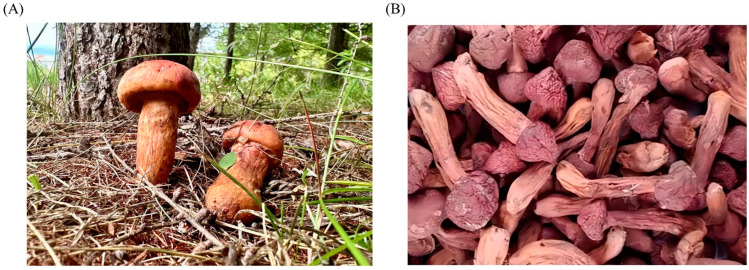
The fruiting body of *C. rutilus*: (**A**) the fresh fruiting body of *C. rutilus* collected from the Greater Khingan Mountains region of Heilongjiang Province, China; (**B**) the dry fruiting body of *C. rutilus*.

The fruiting body of *C. rutilus* (Figure 1) is generally small. When it is first formed, it is generally bell-shaped or nearly cone-shaped. Later, it spreads out slowly, with a slight upward bulge in the middle and a light brown color. The flesh of the fungus is often a little red, and after drying, it is purplish-red. The fungus folds grow outwards along the base of the stipe. The stipe is 6–10 cm long and 1.5–2.5 cm thick, and the stipe tapers down and is solid [32] The growth conditions of *C. rutilus* are harsh, and it grows in the shady slopes with an altitude of 500–700 m. In summer and autumn, the solitary or gregarious *C. rutilus* can often be seen in the pine forest [33]. *C. rutilus*, as a precious understory resource, is common in the north temperate zone and mainly distributed in the pine forests northeast and southwest of China [34].

The pileus of *C. rutilus* is initially bell-shaped and gradually widens into a convex hemisphere. Later, it may become flat and slightly concave in the center, and its diameter is usually between 2 and 6 cm. The surface of the cap is smooth and glossy, there may be reddish-brown and pink fluff, and the edge may be slightly curled. The color of the cover is usually reddish-brown to orange-red, and sometimes it may be slightly orange-yellow [35]. The gill folds are widely spaced, and the common folds are attached to the lid and do not extend to the stem. The color of the pleats is similar to that of the cap but slightly lighter, usually reddish-brown to orange-red [36]. The stipe of *C. rutilus* is usually slender and erect, with a length of 4–10 cm and a diameter of about 0.5–1 cm. The surface of the stem has a rivet-like texture, hence the scientific name. The color of the stem is often red or orange-red, and sometimes it may gradually change to yellow towards the base [29]. The spores of *C. rutilus* are large, orange to brown, and oval in shape, and the color of the sporophores is usually brown [37].

## 3. Fungal Chemical Characteristics of *C. rutilus*

### 3.1. Primary Metabolites of C. rutilus

*C. rutilus* is rich in primary metabolites, including proteins (12.3 g/100 g), eight essential amino acids, minerals (Ca, Fe, K, Na, etc.) [38], carbohydrates, fatty acids [39,40], polysaccharides (6–34 g/100 g), and riboflavin [41]. The silicified extract of *C. rutilus* contains 19 components. Xylitol (56.88%), glucitol (11.08%), fumaric acid (10.92%), and mannitol (6.79%) were identified as the major compounds [42]. The methylated extract contains 13 fatty acids, of which linoleic acid (41.61%) and oleic acid (35.87%) are the main fatty acids [42]. After extracting *C. rutilus* with different substances, it was concluded that hexane extract (IC_50_, 2.22 ± 0.13 μg/mL) and ethyl acetate extract (IC_50_, 2.28 ± 0.18 μg/mL) showed good lipid peroxidation inhibitory activity, while methanol extract showed good butyl cholinesterase inhibitory activity (IC_50_, 45.5 ± 1.1 μg/mL). This biological activity is mainly attributed to polyols [42,43].

### 3.2. Secondary Metabolites of C. rutilus

*C. rutilus* contains different secondary metabolites (Table 1) including 5a, 8a-epidioxyergosta-6,22-dien-3b-ol, ergost-5, 24, (28)-diene-3b,7a,16b-triol, (24R)-ergost-5,7,22- triene-3b-ol, 3b,5a,9a-trihydroxy-(22E,24R)-ergosta-7,22-dien-6-one, 3b,5a-dihydroxy-(22E,24R)-ergosta-7,22-dien-6-one, 5a-ergosta-7,22-dien-3-one, ergosta-4,6,8(14),22tetraene-3-one, 5a-ergost-7-en-3b-ol, 4-hydroxybenzaldehyde, (4-hydroxyphenyl) acetic acid, methyl (4-hydroxyphenyl) acetate, butyl (4 hydroxyphenyl) acetate, butyl (4-hydroxyphenyl) acetate, 3-(3,4-dihydroxyphenyl)-2-propenoic acid, uracil, uridine, adenosine, a-methyl D- xyloside, D-ribonolactone, 6-hydroxy-5,7, dimethoxycoumarin, 7-hydroxycoumarin, esculetin, scopoletin, scoparone, fraxetin, 6,7,8- trimethoxycoumarin, phytodolor, 5,7-dimethoxycoumarin, fraxidin, and esculin [44]. Thus, it can be seen that *C. rutilus* is an important source of dietary fiber, natural anticancer, and antioxidant substances [45,46].

The main phytochemicals of *C. rutilus* are listed in Table 2, where these phytochemicals process diverse bioactivities, such as antioxidant, anti-fatigue, immunomodulatory, anti-tumor, anti-sugar, anti-hyperlipidemic, and gastric protective properties. *C. rutilus* can prevent lipid peroxidation and free radical damage due to its high antioxidant activity [71]. More specifically, the polysaccharides and phenolic compounds in *C. rutilus* have significant antioxidant activity [46], which can neutralize the free radicals and reduce the damage of oxidative stress to the body [72]. These compounds can scavenge the free radicals, enhance the activity of antioxidant enzymes, and adjust the redox balance [73,74]. The polysaccharides and phenolic compounds in *C. rutilus* showed certain anticancer activity [75]. These compounds can inhibit the growth and spread of tumor cells and induce programmed tumor death [76]. They can also regulate the function of the immune system and enhance the body’s immune surveillance of tumors [77]. In addition, the triterpenoids in *C. rutilus* also have anti-angiogenesis effects [78] by blocking the blood supply to tumors. The polysaccharides and phenolic compounds in *C. rutilus* have certain anti-fatigue effects [79]. They can improve the body’s energy metabolism, increase muscle endurance and adaptability, and reduce fatigue [80]. These compounds can also regulate the body’s immune function, reduce the inflammatory response after exercise, and promote the body’s recovery and repair [16].

## 4. Biological Activity of *C. rutilus*

### 4.1. Formation and Function of Antioxidant Activity

Polyphenols have multiple hydroxyl structures, which makes them excellent free radical scavengers [81]. *C. rutilus* contains active compounds such as 4-hydroxybenzaldhyde, (4-hydroxyphenyl) acetic acid, methyl (4-hydroxyphenyl) acetate, 3-(3,4-dihydrohydroxyphenyl)-2-propenoic acid, scopoletin, fraxetin, and esculin [44]. Among them, 4-hydroxybenzaldhyde and the coumarins scopoletin, fraxetin, and esculin can prevent oxidation by scavenging the free radicals [47,51,52,53]. At the same time, in other studies, (4-hydroxyphenyl) acetate was selectively inhibited by the tumor necrosis factor (TNF)-α-induced levels of the redox-sensitive genes, vascular cell adhesion molecule-1, and monocyte chemoattractant protein-1, inhibiting oxidation [48]. Methyl (4-hydroxyphenyl) acetate can reduce the degree of oxidation by inhibiting phenylhydrazine-induced hemolysis of radicals to scavenge most of the free radicals [49]. Currently, there is no literature to directly verify that the antioxidant activity of *C. rutilus* is produced by the above chemical components, but since these components can play an antioxidant role in fungi, *C. rutilus* may also have good antioxidant capacity, which requires further research to verify. Specifically, polyphenols react with free radicals via a redox reaction, thereby reducing the reactivity of free radicals and protecting the cells from oxidative damage [82].

In fungi, coumarins have a good antioxidant capacity [83], and *C. rutilus* also contains these substances, so *C. rutilus* is likely to have this ability. Polysaccharide compounds have a variety of biological activities, including antioxidant, anti-inflammatory, and immunomodulatory activities [84]. Scopoletin can activate some key antioxidant enzymes, such as superoxide dismutase (SOD), glutathione peroxide (GPX), and glutathione-*S*-transfer (GST), to enhance the antioxidant defense system of the cells [51]. This substance has also been found in *C. rutilus* [44], which may reduce the damage caused to the cells by oxidative stress.

*C. rutilus* is rich in vitamin C and vitamin E [85], which may also contribute to the antioxidant capacity of *C. rutilus*. As water-soluble and fat-soluble antioxidants, vitamin C and vitamin E can scavenge the free radicals, inhibit the generation of free radicals in the oxidation reaction chain, and protect the cells from oxidative damage [86]. In addition, *C. rutilus* is rich in some trace elements such as zinc, calcium, and iron [42,87]. As cofactors of antioxidant enzymes, these elements participate in the activity of antioxidant enzymes in the cells, thus enhancing the antioxidant defense capacity of the cells [88].

Moreover, the 2-methoxyadenine nucleoside was isolated and purified from the fruiting body of *C. rutilus* by Feng et al. in 2014 [39], which was determined to have a strong antioxidant capacity (EC_50_ is 0.06 mg/mL). In 2018, Sun et al. [31] found that the crude polysaccharide of *C. rutilus* has a good reducing ability, which was then further separated and purified to obtain two polysaccharide components (GRMPW and GRMPS). The activity results showed that both of them had strong free radical scavenging ability. In the 2016 study, Zhang et al. [89] prepared the total flavonoids of *C. rutilus* via macroporous resin and determined its antioxidant effect. Its ability to scavenge DPPH and the hydroxyl radical (IC_50_ is 0.01 and 0.17 mg/mL) is stronger than that of positive medicine vitamin C. In conclusion, as a fungus rich in antioxidant compounds, the antioxidant properties of *C. rutilus* can be attributed to the synergistic effect of polyphenols, polysaccharides, vitamins, and trace elements.

In determining the redox state, we can evaluate its regulatory effect on the redox state in vivo by measuring the changes in the redox indices in the *C. rutilus* sample, such as the ratio of glutathione (GSH)/oxidized glutathione (GSSG) and the ratio of NADH/NAD^+^ [90]. Using cellular models, such as the oxidative stress model in the cell culture, the protective effect of the *C. rutilus* sample on the cells was observed. Through the animal model, the influence of the *C. rutilus* sample on oxidative stress in animals was observed and the antioxidant activity of *C. rutilus* was comprehensively evaluated [91,92], revealing its antioxidant mechanism and effect.

Free radicals are highly active molecules produced by oxidation processes [93] that can cause cellular damage and oxidative stress [94]. The active components of *C. rutilus* can enhance the activities of antioxidant enzymes such as superoxide dismutase, glutathione peroxidase, and catalase in vivo [45,71]. These enzymes can further scavenge the free radicals in the body [95] and protect the cells from oxidative stress. Polysaccharides can enhance the activities of superoxide dismutase and glutathione peroxidase, reduce oxidative stress, improve mitochondrial function, and reduce the release of free radicals produced by mitochondria [96,97]. The polyphenolic compounds provide electron donors that help restore the reduced state and maintain redox balance [98]. Phenolic compounds also have anti-inflammatory effects and can reduce oxidative stress caused by inflammatory responses [99]. *C. rutilus* was also found to be rich in active compounds such as 4-hydroxybenzaldehyde, (4-hydroxyphenyl) acetic acid, methyl (4-hydroxyphenyl) acetate, 3-(3,4-dihydroxyphenyl)-2-propenoic acid, scopoletin, fraxetin, and esculin [44]. Further detailed experimental evidence is required to demonstrate the biological activity of *C. rutilus*.

Through these mechanisms, the antioxidant activity of *C. rutilus* helps prevent and alleviate the occurrence and development of many chronic diseases, including cardiovascular diseases, neurodegenerative diseases, and diabetes [45,100]. The generation and function of antioxidant activity make *C. rutilus* a potential natural antioxidant and health food [101]. The generation and function of antioxidant activity in *C. rutilus* can be studied using a few analytical methods, including free radical scavenging ability analysis, antioxidant enzyme activity analysis, redox state analysis, and cell and animal experiments [91,92,102,103].

### 4.2. Anti-Tumor Activity of C. rutilus

Abnormal cell proliferation caused by oxidative stress, inflammation, and immune response is related to the occurrence of many tumors [76]. Some fungi may contain compounds with antioxidant, anti-inflammatory, immunomodulatory, and anti-tumor activities, which can improve the immune system and reduce the risk of tumor occurrence [24].

*C. rutilus* has been extensively studied and is considered to have anti-tumor activity [40]. *C. rutilus* can inhibit the growth of tumor cells, and its extract can inhibit a variety of cancer cell lines, including lung cancer, liver cancer, and colon cancer [104]. The active compounds in *C. rutilus* can induce the apoptosis of tumor cells, which is an important cell self-destruction mechanism [105]. *C. rutilus* extract can increase the expression of apoptosis-related proteins such as Bax and caspase-3, while decreasing the expression of Bcl-2, thus promoting the apoptosis of cancer cells [106]. *C. rutilus* was found to have anti-angiogenic properties [107]. Tumor angiogenesis is a key process in tumor growth and metastasis [108]. *C. rutilus* contains 5a, 8a-epidioxyergosta-6,22-dien-3b-ol, 3b, 5a-dihydroxy-(22E,24R)-ergosta-7,22-dien-6-one, 6-hydroxy-5,7-dimethoxycoumarin, 5,7-dimethoxycoumarin, 7-dimethoxycoumarin, ergosta-4,6,8(14),22-tetraene-3-one, phytodolor, and esculetin [44]. Most of these chemical constituents are coumarin and ergostane, and these chemical constituents have been shown to have inhibitory effects on tumors through different mechanisms in other fungi. Among them, 5a, 8a-epidioxyergosta-6,22-dien-3b-ol, 3b, 5a-dihydroxy-(22E,24R)-ergosta-7,22-dien-6-one, and 6-hydroxy-5,7-dimethoxycoumarin have anti-OECM-1 tumor activity [55,56]. 5,7-Dimethoxycoumarin can stimulate melanogenesis and inhibit melanoma by inhibiting Mek 1/2 kinase activity [57]. Ergosta-4,6,8(14) and 22-tetraen-3-one induced G2/M cell cycle arrest and apoptosis in human hepatocellular carcinoma HepG2 cells, which reduced the incidence of liver cancer and the growth rate of liver cancer cells [62]. 6-Hydroxy-5,7-dimethoxycoumarin and 5,7-dimethoxycoumarin inhibited the growth of Jurkat tumor cells and A-375 tumor cells, respectively, and played an anti-tumor role [63,65]. Phytodolor inhibited the intracellular content of TNF-α and PTGS2 proteins and the expression of TNF-α and PTGS2 genes and inhibited the apoptosis of LPS-activated human monocytes in the absence of serum. In addition, phytodolor inhibited the translocation of the p65 subunit of redox-regulated NF-κB in the nucleus of LPS-activated human macrophages. It plays an anti-tumor role [69]. Esculetin has a cytotoxic effect on the leukemia cell line HL-60 and plays an anti-tumor role [70]. At present, there is no literature to directly verify that these chemical components in *C. rutilus* have the same effect, but since these components can play an anti-tumor role in fungi, *C. rutilus* may also have good anti-tumor ability, which needs further experimental research to verify. *C. rutilus* extract can promote the activation and proliferation of immune cells [80], These results confirmed that *C. rutilus* has anti-tumor activity.

### 4.3. Immunomodulatory Activity of C. rutilus

The immune system is an important part of maintaining the health of the body and acts as a line of defense against pathogens and abnormal cells [109]. Immunoregulation is a key process in the immune system that can balance the immune response, regulate the activity of immune cells, and maintain immune balance [110].

**Figure 2:**
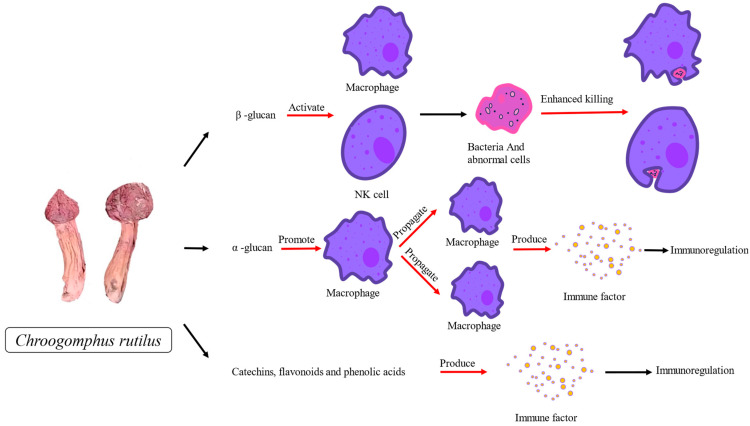
The summary of the mechanism of the immunomodulatory activity of *C. rutilus*.

The active components in *C. rutilus* can regulate the activation and proliferation of immune cells (Figure 2). Adenosine and scoparone can inhibit T cell proliferation, cytokine production and cytotoxicity, and NK cell toxicity in other fungi), NKT cytokine production and CD40L upregulation, antigen presentation and cytokine production by macrophages or dendritic cells, and neutrophil oxidative burst activity. These cytokines play an important role in immune regulation and inflammation [111]. It also inhibits the human monocyte response to phytohaemagglutinin and mixed lymphocyte reaction and can be used to combat transplant rejection and autoimmune diseases [58,59]. *C. rutilus*, which plays an immunomodulatory role, also contains these two substances. At present, no experiment directly proves that the antioxidant activity of *C. rutilus* is produced by these two chemical components, but *C. rutilus* probably also has good immunomodulatory ability, which needs more research to verify.

The immunomodulatory activity of *C. rutilus* is closely related to the synergistic effects of polysaccharides (such as *β*-glucan and *α*-glucan), polyphenols (such as catechins, flavonoids, and phenolic acids), and antioxidants (such as vitamin C and vitamin E) [112,113,114]. Polysaccharides in *C. rutilus* are considered to be one of the important components of its immunomodulatory activity. It has been found that the polysaccharide compounds in *C. rutilus* mainly include *β*-glucan and *α*-glucan [46]. These polysaccharide compounds can stimulate the activation and proliferation of immune cells and enhance the ability of immune cells to produce cytokines [115]. For example, *β*-glucan can activate macrophages and natural killer cells and increase their killing effect on pathogens and abnormal cells [116]. In addition, *α*-glucan has an immunomodulatory effect, which can promote the proliferation of immune cells and the production of immune factors, thus enhancing the immune response [117]. The polyphenols in *C. rutilus* also play an important role in its immunomodulatory activity. The polyphenols found in *C. rutilus* are mainly catechins, flavonoids, and phenolic acids [42]. These compounds can regulate the balance of the immune response by regulating the activity of immune cells and the production of cytokines. For example, catechins have immunomodulatory effects that can regulate the function of immune cells and the production of cytokines [118].

In addition, antioxidants such as vitamin C and vitamin E are also involved in its immunomodulatory activity [119,120]. Vitamin C and vitamin E can reduce oxidative stress damage to immune cells and regulate the activity of immune cells and the immune response [121].

### 4.4. Anti-Fatigue Activity of C. rutilus

Fatigue is a common physical and psychological condition in modern society, which has a negative impact on individual health and the quality of life. As a natural herbal resource, *C. rutilus* has been extensively studied and shows potential anti-fatigue activity.

*C. rutilus* extract has an anti-fatigue effect and can delay the onset and development of fatigue. This activity is related to the synergistic effect of polysaccharides, polyphenols, and other bioactive substances in *C. rutilus* [122]. Polysaccharide compounds in *C. rutilus* are considered to be one of the important components of its anti-fatigue activity [122]. Crude polysaccharide compounds can provide energy supply and regulate energy metabolism, thus increasing the endurance and anti-fatigue ability of the body [79]. Polysaccharide compounds in *C. rutilus* have been found to improve the endurance of physical activity, reduce fatigue, and promote recovery [113,122].

Additionally, its anti-fatigue activity also includes the participation of polyphenols [113]. Polyphenols, such as the catechins and flavonoids abundant in *C. rutilus*, are believed to exhibit a beneficial anti-fatigue effect [123]. There are other bioactive substances in *C. rutilus*, such as vitamins, minerals, and amino acids [45], which may also play a role in anti-fatigue. Vitamins and minerals are involved in the regulation of energy metabolism and muscle function, which helps to improve the body’s endurance and anti-fatigue ability [124]. Amino acids are the basic units of protein, which play an important role in maintaining normal muscle function and repairing damaged tissue [125]. In 2014, animal experiments were conducted to investigate the function of the *C. rutilus* polysaccharide in alleviating physical fatigue. Mice were given low (100 mg/kg/d), medium (250 mg/kg/d), and high (625 mg/kg/d) doses of the *C. rutilus* polysaccharide, and exhaustive swimming time, blood lactic acid, and blood urea were measured. The results showed that the effect of the mid-dose group was significant. Compared with the control group, the middle dose group of *C. rutilus* polysaccharide can significantly reduce the content of blood urea nitrogen and blood lactic acid and increase the glycogen storage in the body, indicating that the *C. rutilus* polysaccharide has a certain function of alleviating physical fatigue [75].

### 4.5. Hypoglycemia Activity of C. rutilus

Polysaccharide compounds have biological activities such as lowering blood glucose, improving islet function, and increasing insulin sensitivity [126]. These polysaccharide compounds may block the process of sugar digestion and absorption by interacting with glycosidase in the gut, thereby lowering the blood glucose levels [127].

In addition, fraxidin has been shown to inhibit aldose reductase activity and platelet aggregation, inhibit the rise of the blood sugar levels in the human body, and may play an anti-hyperglycemia role [52]. *C. rutilus* contains this chemical, which may play an anti-hyperglycemic role. However, there is no evidence that the anti-hyperglycemia effect of *C. rutilus* comes from fraxidin, which needs further research to prove. Further research has also shown that *C. rutilus* may exert its anti-sugar activity by regulating the insulin signaling pathway and improving insulin sensitivity [128]. These compounds may also affect the activities of key enzymes involved in glucose metabolism and promote glucose utilization and energy metabolism [128].

### 4.6. Gastric Protective Activity of C. rutilus

**Figure 3:**
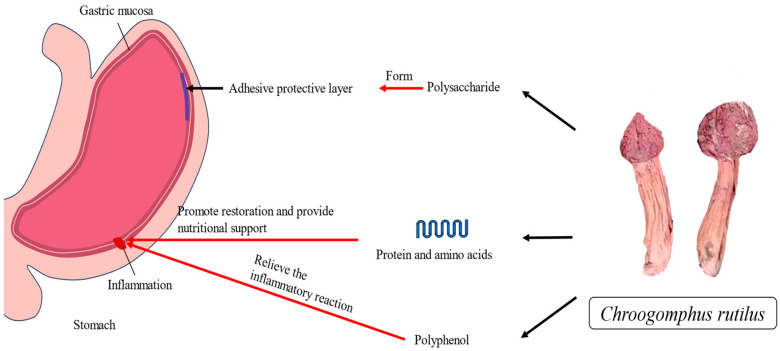
The summary of the mechanism of gastric protective activity of *Chroogomphus rutilus*.

Studies have shown that *C. rutilus* has significant gastric protective activity (Figure 3), which has a positive effect on protecting the gastric mucosa, inhibiting gastric ulcers, and relieving gastric inflammation. The active constituents of *C. rutilus* are involved in its gastric protective activity. The polysaccharide compounds were found to be one of the important components of its gastric protective activity [129]. Polysaccharide compounds have a variety of biological activities, including anti-inflammatory, antioxidant, and mucosal protection [130]. *C. rutilus* contains 6,7,8-trimethoxycoumarin, and 6,7,8-trimethoxycoumarin can improve the gastric protective activity of GU induced by hydrochloric acid or ethanol and indomethacin, resulting in the reversal of GU by more than 90%. As a medicinal mushroom, *C. rutilus* probably also has gastric protective biological activity, which requires further studies to validate [44,64]. This improvement in gastric protective activity has been confirmed in other fungi. At the same time, the polysaccharide compounds in the plant can reduce the inflammatory response of the gastric mucosa and promote the repair and regeneration of the gastric mucosa [131].

In addition, the polyphenols also play an important role in its gastric protective activity. Polyphenols have antioxidant and anti-inflammatory effects, which can reduce the oxidative stress and inflammatory response of the gastric mucosa and protect the gastric mucosa from damage [99]. Moreover, protein, amino acids, vitamins, etc., are also involved in gastric protective activities. Protein and amino acids can provide nutrients needed for gastric mucosal repair and promote gastric mucosal recovery. Vitamins participate in the metabolism and repair of mucosal tissues and play an important role in gastric mucosal health [132,133]. *C. rutilus* may have good gastric protective activity because of the meaning of these substances, but this needs more follow-up experiments to verify.

### 4.7. Hypolipidemic Activity of C. rutilus

The active constituents of *C. rutilus* play an important role in its anti-hyperlipidemic activity. Polysaccharide compounds can regulate lipid metabolism and inhibit cholesterol synthesis [134]. Polysaccharide compounds can regulate blood lipid metabolism in many ways, including reducing the levels of total cholesterol, low-density lipoprotein cholesterol (LDL-C), and triacylglycerol, increasing the levels of high-density lipoprotein cholesterol (HDL-C) and promoting cholesterol excretion [135,136]. These effects help reduce the blood lipid levels and lipid accumulation in the blood, thus playing an anti-hyperlipidemic role.

Polyphenols have antioxidant activity, which can reduce lipid peroxidation and prevent the formation of lipid oxidation products, thus reducing the blood lipid levels [86]. Protein, amino acids, and dietary fiber can also regulate blood lipid metabolism and promote the balance of blood lipid metabolism [135]. Further studies have also shown that the anti-hyperlipidemic activity of *C. rutilus* is closely related to the regulation of relevant enzymes. For example, the active components can inhibit key enzymes in cholesterol synthesis, such as HMG-CoA reductase, resulting in reduced cholesterol synthesis [135]. In addition, the components of *C. rutilus* may also affect the expression of the genes involved in lipid metabolism, further regulating the balance of lipid metabolism [113,137]. The 5a-ergosta-7,22-dien-3-one contained in *C. rutilus* can be used as an (exo-α-sialidase) inhibitor and an antifungal agent, which plays an immunomodulatory role. Hydroxyl groups can separate polar lipids and reduce blood lipids and cholesterol. In other experimental studies, 5a-ergosta-7,22-dien-3-one has been proven to play a role in reducing blood lipids [44,61]. Another medicinal mushroom, *C. rutilus*, may also have good anti-hyperlipidaemic activity, which can be tested in subsequent experimental studies.

### 4.8. Neuroprotective Effects of C. rutilus

The crude polysaccharide has a protective effect against neuronal damage. Nervous system diseases such as Alzheimer’s disease, Parkinson’s disease, and stroke are usually associated with oxidative stress, inflammatory response, and neuronal damage [138,139] Polysaccharide compounds can regulate neuronal metabolic activity, improve nerve conduction and cell signaling, and promote neuronal survival and repair [140]. Studies have shown that *C. rutilus* and its extracts have significant activity in neuronal protection [46]. In addition, *C. rutilus* can also inhibit the inflammatory response and reduce cell death caused by neuronal inflammatory injury [46]. These protective effects may be related to the mechanism by which the active components of *C. rutilus* regulate the cell signaling pathways, reduce the release of inflammatory mediators, and promote the self-repair ability of the cells [114,135]. *C. rutilus* may have neuroprotective activity, and uridine can be phosphorylated to nucleotides for DNA and RNA synthesis and the synthesis and glycosylation of membrane components. Uridine nucleotides and UDP sugars can be released from the neurons and glial cells. It is used as a neuroprotective agent in the treatment of neurodegenerative diseases [60]. However, *C. rutilus* contains uridine, which may have a neuroprotective effect, but this has not been confirmed by any direct experiments and further research is needed to prove this.

## 5. Conclusions

Due to the taste and nutritional value of *C. rutilus*, people’s demand for *C. rutilus* is increasing. *C. rutilus* is a fungus with potential medicinal value that has been extensively studied and found to have various biological activities. Due to its medicinal value, *C. rutilus* is also used in medicine to treat various diseases. As a species of medicinal mushroom, *C. rutilus* has shown a wide application prospect in clinical research. The properties of its active components, such as anti-tumor, cardiovascular protection, immunomodulation, anti-inflammation, anti-oxidation, and anti-aging, provide a basis for its further research and development in the fields of tumor treatment, cardiovascular diseases, immunomodulation, and anti-aging. However, further clinical research and practice are needed to verify its safety and efficacy and to promote its use in clinical practice. Due to its antioxidant, anti-tumor, immunomodulatory, anti-fatigue, anti-sugar, gastric protective, anti-hyperlipidaemic, and neuronal protective activities, *C. rutilus* has attracted a lot of attention in the fields of biochemistry and pharmacology. At the same time, the mechanism behind some of the medicinal activities has hardly been studied. Therefore, future research must pay attention to these potential mechanisms. Finally, despite the remarkable pharmacological effects of *C. rutilus* in vitro and in vivo, there is a lack of information on its safe use, therapeutic index, and risk–benefit ratio in humans. These data are very useful for the development of new pharmaceutical preparations based on the active principles of *C. rutilus*.

## Figures and Tables

**Table 1:** The secondary metabolites of *C. rutilus*.

Components	Classification	Structure	Function	References
4-Hydroxybenzaldehyde	hydroxybenzaldehyde	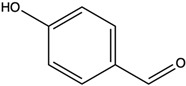	Scavenged free radicals and promoted antioxidation	[47]
(4-hydroxyphenyl) acetic acid	monocarboxylic acid	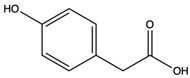	Selectively inhibited tumor necrosis factor (TNF)-α-inducible levels of the redox-sensitive genes, vascular cell adhesion molecule-1, and monocyte chemoattractant protein-1	[48]
Methyl (4-hydroxyphenyl) acetate	methyl ester	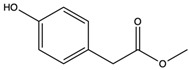	Inhibited phenyl hydrazine-induced hemolysis of erythrocytes to scavenge most of the free radicals generated	[49]
3-(3,4-Dihydroxyphenyl)-2-propenoic acid	monocarboxylic acid	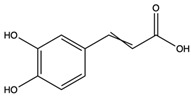	Scavenged free radicals and promoted antioxidation	[50]
Scopoletin	coumarin	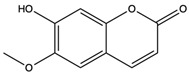	Scavenged free radicals and promoted antioxidation. Activated some key antioxidant enzymes, such as superoxide dismutase (SOD), glutathione peroxidase (GPx), and glutathione -S- transferase (GST) to enhance the antioxidant defense system of cells	[51]
Fraxetin	coumarin	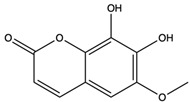	Scavenged free radicals and promoted antioxidation	[52]
Esculin	coumarin	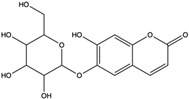	Scavenged free radicals and promoted antioxidation	[53]
5a, 8a-Epidioxyergosta-6, 22-dien-3b-ol	ergosterol peroxide	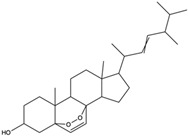	Induced a cytotoxic effect on the OECM-1 cell strain and exerted an anti-tumor role	[54]
3b,5a-Dihydroxy-(22E, 24R)-ergosta-7,22-dien-6-one	ergostanoid	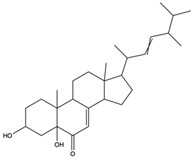	Exerted a cytotoxic effect on the MCF-7 cell strain and fulfilled an anti-tumor role	[55]
6-Hydroxy-5,7-dimethoxycoumarin	coumarins	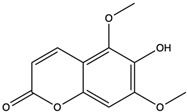	Induced a cytotoxic effect on the L1210 cell strain and played an anti-tumor role	[56]
5,7-Dimethoxycoumarin	coumarins	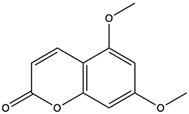	Inhibited Mek 1/2 kinase activity and stimulated melanin production to inhibit melanoma	[57]
Adenosine	nucleoside	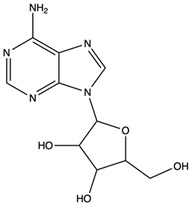	Inhibited the activities of T cells (proliferation, cytokine production, and cytotoxicity), NK cells (cytotoxicity), NKT cells (cytokine production and CD40L up-regulation), macrophages/dendritic cells (antigen presentation and cytokine production), and neutrophils (oxidative burst)	[58]
Scoparone	coumarins	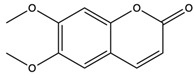	Suppressed the responses of human mononuclear cells to phytohemagglutinin and mixed lymphocyte reaction for use against transplantation rejection and autoimmune disease	[59]
Uridine	nucleoside	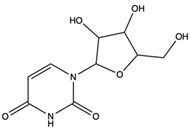	Uridine is phosphorylated into nucleotides for the synthesis of DNA and RNA, as well as the synthesis of membrane components and glycosylation. Uridine nucleotides and UDP sugars may be released from neurons and glial cells. Used as neuroprotective agent for treating neurodegenerative diseases	[60]
5a-Ergosta-7,22-dien-3-one (Stellasterin)	lanostanoids	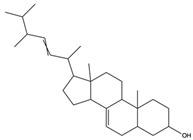	(External α-sialidase) inhibitors and antifungal agents, which play an immunomodulatory role. Hydroxyl groups can separate polar lipids and reduce blood fat and cholesterol	[61]
Ergosta-4,6,8(14),22tetraene-3-one	ergostanoid	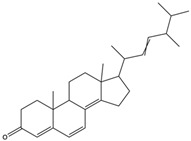	Induced G2/M cell cycle arrest and apoptosis in human hepatocellular carcinoma HepG2 cells	[62]
6-Hydroxy-5,7-dimethoxycoumarin (Fraxinol)	coumarins	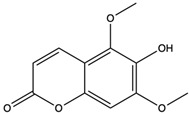	Inhibited the growth of Jurkat cell line tumor cells	[63]
6,7,8-Trimethoxycoumarin	coumarins	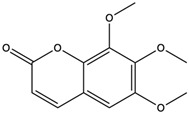	The gastric protective activity of GU induced by HCl/ethanol and indomethacin was improved, resulting in more than 90% reversal of GU	[64]
5,7-Dimethoxycoumarin (citropten)	coumarins	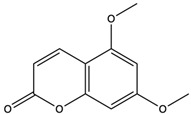	Inhibited the growth of A-375 melanoma cells	[65]
Fraxidin	coumarins	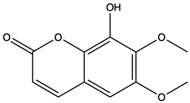	Exerted inhibitory effects towards aldose reductase activity and platelet aggregation	[66]
Scopoletin	coumarins	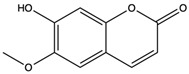	Scopoletin has obvious anti-inflammatory activity in inhibiting the overproduction of PGE2 and TNF-α and neutrophil infiltration	[67]
7-Hydroxycoumarin	coumarins	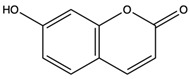	Inhibited [3H]thymidine, [3H]uridine, and [3H]leucine incorporation. Inhibited the intracellular production of prostate-specific antigen by LNCaP cells. Have direct antitumor (cytostatic) activity as well as immunomodulatory activity	[68]
Phytodolor	coumarins	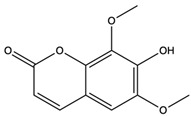	The intracellular content of TNF-α and PTGS2 protein and the expression of TNF-α and PTGS2 gene were inhibited, and the induced apoptosis of LPS-activated human monocytes was inhibited in the absence of serum. In addition, phytodolor inhibited the translocation of p65 subunit of redox-regulated NF-κB in LPS-activated human macrophage nuclei. Played an anti-tumor role	[69]
Esculetin	coumarins	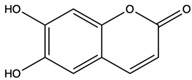	Exerted a cytotoxic effect on the Leukemia HL-60 cell strain and fulfilled an anti-tumor role	[70]
3b,5a,9a-Trihydroxy-(22E,24R)-ergosta-7,22-dien-6-one	ergostanoid	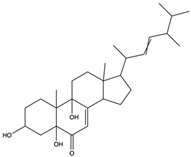	/	/
5a-Ergosta-7-en-3b-ol	ergostanoid	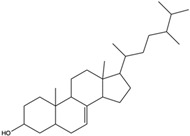	/	/

**Table 2:** The major bioactive components from *C. rutilus* and their biological activities.

Compounds or Extracts	Biological Activity	Method	References
2-Methoxyadenine nucleoside, flavone	Antioxidant activity	Separating and purifying fruiting bodies and preparing macroporous resin	[39]
Polysaccharide	Antifatigue activity	Mice were given low (100 mg/kg/d), medium (250 mg/kg/d), and high (625 mg/kg/d) doses of Pleurotus eryngii polysaccharide	[75]
Polysaccharides (*β*-glucan and *α*-glucan)	Immunomodulatory activity	Organic extraction	[46]
Crude extract	Antitumor activity	Organic extraction (95% ethanol)	[45]

## Data Availability

Not applicable.
